# Supplementary material for: Modulation of oculomotor control during reading of mirrored and inverted texts
Source: Sci Rep. 2020 Mar 6;10:4210. doi: 10.1038/s41598-020-60833-6 (PMC7060224; doi:10.1038/s41598-020-60833-6)
Supplement: Supplementary file 1 — Supplementary Information to: Modulation of oculomotor control during reading of mirrored and inverted texts. [file 41598_2020_60833_MOESM1_ESM.pdf]

Supplementary Information to: Modulation of oculomotor control during reading of mirrored and inverted texts

Johan Chandra*, André Krügel, and Ralf Engbert

University of Potsdam, Germany

December 19, 2019

*To whom correspondence should be addressed:
Johan Chandra
Department of Psychology & Cognitive Science Program
University of Potsdam
Am Neuen Palais 10
14469 Potsdam
Germany
E-mail: johan.chandra@uni-potsdam.de
Phone: +49 331 977-2184, Fax: +49 331 977-2794

1 Method

1.1 Global summary statistics

Statistics of fixation durations and saccade lengths of all valid fixations were computed for a global summary analysis. Words are categorised based on their length (WLC): short (≤ 4 characters), medium (5-7 characters), and long (≥ 8 characters) for further statistical analysis.

Fixation durations. One way to quantify the visual information processing in reading is to examine the time spent on fixating words (fixation duration). Single fixation duration (SFD) refers to the time spent on a word which received exactly one fixation. When a word received more than one fixations in first-pass reading, the duration of the first and second fixations generated the first of multiple fixations duration (FMD) and second of multiple fixation duration (SMD). Inspection of duration distributions suggested that log-transformation is required to meet assumptions of Linear-Mixed Model. Therefore, for word-length effect analysis, the fixation duration measures were transformed into their logarithmic values.

Fixation probability. Fixation probability on a word depends on different saccade types. For first-pass reading reading data, we computed the skipping, refixation and regression probabilities. Regression cases were counted for words whose saccade directions went against reading directions, right to left for control condition and vise versa for experimental conditions. For each word that is the target of skipping saccades and refixations and/or source of regression saccades, a logical value of 1 is assigned to the word. Otherwise, a logical value of 0 was assigned.

The coefficients of the fixed effect of word length classes and the random effect for various participants and sentences were estimated using the lme4 package (Bates et al., 2015). Each independent variable of fixation duration and fixation probability measure is modelled as a function of word length (`WLC`) across sessions (`sess`), with a fully parameterised variance-covariance matrix for participants expressed in the term of `pID`. Furthermore, the variance for the intercept over sentence number (expressed in the terms $(1|\texttt{sID})$) are taken into account. The model also allows us to examine the interaction of word-length effect between the control and experimental sessions. For instance, the log-transformed duration of the first of multiple fixations(`FMD`) is modelled as

$$\texttt{lmer}(\log(\texttt{FMD}) = \texttt{WLC} * \texttt{sess} + (1 + \texttt{WLC} + \texttt{sess}|\texttt{pID}) + (1|\texttt{sID})), \tag{1}$$

Likewise, refixation probability (RFP) is modelled as

$$\texttt{glmer}(\texttt{RFP} = \texttt{WLC} * \texttt{sess} + (1 + \texttt{WLC} + \texttt{sess}|\texttt{pID}) + (1|\texttt{sID})), \tag{2}$$

Note that the models for fixation probabilities were estimated using general function of `glmer()` due to it's ability to statistically analyse data with binary outcome. All models were estimated based on restricted maximum likelihood (REML).

1.2 Initial Landing Position

Within-word landing positions observed during reading are widely distributed and sometimes extend to the neighboring words. Observations of word-based landing positions in reading experiments are typically truncated at word boundaries (McConkie et al., 1988; Engbert & Nuthmann, 2008). To obtain estimations of the means and standard deviations of the landing-position distributions, truncated Gaussian curves for distributions of word-based fixation positions were fitted using Bayesian parameter inference (Kruschke, 2014) available in the package *rjags* (Plummer, 2016) in the R environment.

Only fixations from inter-word forward saccades were considered in the estimation procedure. For each session, fixation data were grouped based on word length (3-7 letters) and launch-site distance (-1 to -5 characters), resulting in three different word-length and launch-site session-specific data subsamples. For each data subset S_i, a two-dimensional posterior distribution was estimated over the parameters mean μ and standard deviation σ of the underlying Gaussian landing-position distribution conforming to

$$p(\mu, 1/\sigma^2|S_i) = \frac{p(S_i|\mu, 1/\sigma^2)p(\mu, 1/\sigma^2)}{\int\int p(S_i|\mu, 1/\sigma^2)p(\mu, 1/\sigma^2)\,\mathrm{d}\mu\mathrm{d}1/\sigma^2}\ . \tag{3}$$

Observations of fixation landing positions are assumed to be generated by a normal-density likelihood function $p(S_i|\mu, 1/\sigma^2)$ with mean μ and precision $\tau = 1/\sigma^2$. With $p(\mu, 1/\sigma^2)$ we specified a normally distributed prior on the mean μ with mean M and precision T and a prior on τ distributed as a gamma density distribution with shape parameter A and rate B (see Kruschke (2014)). The parameters M, T (for the prior over μ) and A,B (for the prior τ) were derived as follows: For the control condition, distributions of landing positions for each word length and launch-site distance were independently fitted by a truncated Gaussian function and the parameters of these fits were used to estimate the parameters of the empirical prior distribution. The estimated parameters from the control condition were used to estimate landing position distributions for the first experimental session and the resulting parameters were used to systematically update the prior distribution for the second experimental session.

2 Tables & Figures

Table A1: Global summary statistics.

Condition		Control	Mirrored Words (mW)			Mirrored Letters (mL)			Inverted Words (iW)			Scrambled Letters (sL)		
Session		0	1	2	3	1	2	3	1	2	3	1	2	3
Detected fixations	N	82487	21598	27500	26957	26677	24205	22246	22531	24104	25728	25549	26648	24062
Discarded fixation	N	40377	10351	12251	11691	12143	11639	11271	10340	10766	10933	11361	11350	10597
	%	48.95	47.93	44.55	43.37	45.52	48.09	50.67	45.89	44.66	42.49	44.47	42.59	44.04
Valid fixation	N	42110	11247	15249	15266	14534	12566	10975	12191	13338	14795	14188	15298	13465
	%	51.05	52.07	55.45	56.63	54.48	51.91	49.33	54.11	55.34	57.51	55.53	57.41	55.96
First-pass fixation	N	32343	6925	11166	11620	11200	10061	8809	9479	10428	11912	10269	10034	8836
	%	76.81	61.57	73.22	76.12	77.06	80.07	80.26	77.75	78.18	80.51	72.38	65.59	65.62
Forward saccades	N	14956	2960	5386	5725	5636	4379	3909	4050	4653	5363	4887	4219	3843
	%	46.24	42.74	48.24	49.27	50.32	43.52	44.38	42.73	44.62	45.02	47.59	42.05	43.49
Skipping saccades	N	9963	516	834	931	1476	2193	1964	431	674	774	1973	1999	1807
	%	30.8	7.45	7.47	8.01	13.18	21.8	22.3	4.55	6.46	6.5	19.21	19.92	20.45
Refixations	N	7357	3436	4933	4957	4082	3486	2930	4989	5096	5772	3400	3805	3170
	%	22.75	49.62	44.18	42.66	36.45	34.65	33.26	52.63	48.87	48.46	33.11	37.92	35.88
Regressions	N	3692	693	808	854	734	695	638	549	584	649	992	985	966
	%	11.42	10.01	7.24	7.35	6.55	6.91	7.24	5.79	5.6	5.45	9.66	9.82	10.93
fixation duration	mean [ms]	245	354	351	337	299	277	266	383	344	329	306	300	304
forward saccade amplitude	mean [char]	7.82	5.19	5.58	5.81	5.53	6.56	6.86	5.25	5.55	5.81	6.18	6.15	6.38
backward saccade amplitude	mean [char]	4.23	2.82	2.97	3.03	3.01	2.88	2.67	2.63	2.57	2.79	4.66	3.95	4.25

Table A2: Results from Linear Mixed-Effects: Single Fixation Duration

Condition		*b*	*SE*	*t value*
Mirrored Letters (mL)	Intercept	5.47	0.04	124.53
	WLC	0.02	0.02	**1.15**
	Session 1	0.18	0.02	9.69
	Session 2	0.10	0.02	5.32
	Session 3	0.09	0.02	4.22
	WLC:Session 1	0.06	0.01	7.79
	WLC:Session 2	0.07	0.01	9.38
	WLC:Session 3	0.06	0.01	8.30
Mirrored Words (mW)	Intercept	5.44	0.04	142.66
	WLC	0.02	0.02	**0.87**
	Session 1	0.31	0.07	4.54
	Session 2	0.31	0.06	5.11
	Session 3	0.29	0.06	5.10
	WLC:Session 1	-0.14	0.01	-12.13
	WLC:Session 2	-0.07	0.01	-7.08
	WLC:Session 3	-0.01	0.01	**-0.99**
Inverted Words (iW)	Intercept	5.48	0.04	128.67
	WLC	0.01	0.02	**0.45**
	Session 1	0.36	0.05	7.48
	Session 2	0.27	0.03	7.84
	Session 3	0.22	0.03	6.17
	WLC:Session 1	-0.21	0.01	-18.10
	WLC:Session 2	-0.10	0.01	-9.56
	WLC:Session 3	-0.06	0.01	-5.71
Scrambled Letters (sL)	Intercept	5.46	0.03	186.58
	WLC	0.01	0.02	**0.24**
	Session 1	0.11	0.04	2.91
	Session 2	0.11	0.05	2.21
	Session 3	0.10	0.04	2.34
	WLC:Session 1	0.10	0.01	11.95
	WLC:Session 2	0.12	0.01	14.61
	WLC:Session 3	0.09	0.01	10.80

Note: Fixation duration value is log-transformed. WLC = word length. Non-significant values are marked in bold.

Table A3: Results from Linear Mixed-Effects model: Duration of First of Multiple Fixations

Conditions		*b*	*SE*	*t value*
Mirrored Letters (mL)	Intercept	5.43	0.05	114.86
	WLC	-0.01	0.02	**-0.31**
	Session 1	0.16	0.03	4.56
	Session 2	0.04	0.03	**1.23**
	Session 3	0.03	0.03	**0.93**
	WLC:Session 1	0.09	0.01	6.04
	WLC:Session 2	0.09	0.02	5.86
	WLC:Session 3	0.08	0.02	4.68
Mirrored Words (mW)	Intercept	5.37	0.05	101.59
	WLC	0.04	0.03	**1.56**
	Session 1	0.34	0.04	9.23
	Session 2	0.32	0.03	10.98
	Session 3	0.24	0.05	4.36
	WLC:Session 1	-0.16	0.02	-10.31
	WLC:Session 2	-0.20	0.01	-14.05
	WLC:Session 3	-0.10	0.01	-6.60
Inverted Words (iW)	Intercept	5.46	0.04	122.19
	WLC	0.03	0.02	**1.22**
	Session 1	0.33	0.05	6.32
	Session 2	0.25	0.05	4.83
	Session 3	0.20	0.05	4.14
	WLC:Session 1	-0.16	0.02	-10.58
	WLC:Session 2	-0.14	0.02	-9.11
	WLC:Session 3	-0.11	0.02	-7.32
Scrambled Letters (sL)	Intercept	5.44	0.03	162.02
	WLC	0.01	0.02	**0.27**
	Session 1	0.06	0.04	**1.54**
	Session 2	0.06	0.03	**1.84**
	Session 3	0.06	0.03	**1.77**
	WLC:Session 1	0.15	0.02	8.80
	WLC:Session 2	0.22	0.02	12.83
	WLC:Session 3	0.15	0.02	8.52

Note: Fixation duration value is log-transformed. WLC = word length. Non-significant values are marked in bold.

Table A4: Results from Linear Mixed-Effects model: Refixation Probabilities

Condition		*b*	*SE*	*z value*	*Pr(>—z—)*
Mirrored Letters (mL)	Intercept	-1.63	0.12	-13.75	0.000
	WLC	1.15	0.09	12.52	0.000
	Session 1	1.29	0.18	7.32	0.000
	Session 2	0.76	0.14	5.53	0.000
	Session 3	0.48	0.17	2.75	0.006
	WLC:Session 1	0.37	0.05	6.86	0.000
	WLC:Session 2	0.41	0.06	7.33	0.000
	WLC:Session 3	0.38	0.06	6.48	0.000
Mirrored Words (mW)	Intercept	-1.35	0.18	-7.62	0.000
	WLC	1.15	0.11	10.50	0.000
	Session 1	1.39	0.23	6.15	0.000
	Session 2	1.35	0.14	9.78	0.000
	Session 3	1.19	0.21	5.71	0.000
	WLC:Session 1	0.55	0.05	10.00	0.000
	WLC:Session 2	0.64	0.05	12.98	0.000
	WLC:Session 3	0.69	0.05	13.85	0.000
Inverted Words (iW)	Intercept	-1.51	0.17	-9.03	0.000
	WLC	1.21	0.07	18.14	0.000
	Session 1	1.87	0.15	12.49	0.000
	Session 2	1.69	0.17	10.06	0.000
	Session 3	1.65	0.17	9.98	0.000
	WLC:Session 1	0.64	0.05	11.76	0.000
	WLC:Session 2	0.52	0.05	9.96	0.000
	WLC:Session 3	0.62	0.05	12.17	0.000
Scrambled Letters (sL)	Intercept	-1.51	0.15	-9.73	0.000
	WLC	1.46	0.10	13.95	0.000
	Session 1	0.78	0.23	3.48	0.000
	Session 2	0.57	0.20	2.90	0.004
	Session 3	0.67	0.18	3.64	0.000
	WLC:Session 1	0.32	0.05	5.92	0.000
	WLC:Session 2	0.42	0.06	7.61	0.000
	WLC:Session 3	0.31	0.06	5.52	0.000

Note: WLC = word length. Non-significant values are marked in bold.

Table A5: Results from Linear Mixed-Effects model: Effect sizes accross manipulation types

Measures		*b*	*SE*	*z value*	*Pr(>—z—)*
Single Fixation Duration (SFD)	Intercept	5.65	0.03	213.36	
	WLC	-0.03	0.01	-3.57	
	mL	0.09	0.02	3.95	
	sL	0.01	0.02	**0.46**	
	mW	0.05	0.01	3.90	
	iW	0.04	0.01	4.07	
	WLC:mL	0.03	0.00	6.97	
	WLC:sL	0.02	0.00	8.38	
	WLC:mW	-0.05	0.00	-19.63	
	WLC:iW	-0.04	0.00	-19.74	
First of Multiple Fixations duration (FMF)	Intercept	5.60	0.03	212.41	
	WLC	0.00	0.01	**-0.29**	
	mL	0.08	0.02	4.08	
	sL	-0.01	0.01	**-0.42**	
	mW	0.06	0.01	5.47	
	iW	0.04	0.01	4.45	
	WLC:mL	0.04	0.01	5.82	
	WLC:sL	0.05	0.01	9.21	
	WLC:mW	-0.06	0.00	-17.12	
	WLC:iW	-0.04	0.00	-14.35	
Refixation Probability (RFX)	Intercept	-0.40	0.08	-5.03	0.000
	WLC	1.59	0.04	36.17	0.000
	mL	0.62	0.08	7.33	0.000
	sL	0.04	0.06	0.66	**0.506**
	mW	0.21	0.04	4.80	0.000
	iW	0.21	0.04	5.91	0.000
	WLC:mL	0.17	0.02	7.17	0.000
	WLC:sL	0.08	0.02	4.47	0.000
	WLC:mW	0.07	0.01	5.05	0.000
	WLC:iW	0.05	0.01	4.54	0.000

Note: LMM model with helmert contrast. SFD and FMD values are log-transformed (base 10).

WLC = word length. Non-significant values are marked in bold.

Table A6: Quadratic fit to initial landing position curve (two-fixation cases) for reading normal text: Estimates of parameters A, B and C

Session	Word Length	Parameters	Estimate	Std. Error	z value	Pr(z)
0	4	A	0.051	0.020	2.466	0.013
	4	B	0.065	0.008	7.814	0.000
	4	C	2.528	0.101	24.916	0.000
	5	A	0.023	0.015	1.497	0.134
	5	B	0.040	0.004	9.139	0.000
	5	C	3.336	0.121	27.468	0.000
	6	A	0.019	0.012	1.516	0.130
	6	B	0.023	0.003	8.859	0.000
	6	C	4.21	0.168	25.043	0.000
	7	A	-0.008	0.016	-0.518	0.604
	7	B	0.018	0.003	7.115	0.000
	7	C	4.98	0.251	19.834	0.000

Table A7: Quadratic fit to initial landing position curve (two-fixation cases) for reading ***mirrored-word (mW)*** text: Estimates of parameters A, B and C

Session	Word Length	Parameters	Estimate	Std. Error	z value	Pr(z)
1	4	A	0.080	0.022	3.684	0.000
	4	B	0.048	0.009	5.309	0.000
	4	C	2.670	0.173	15.650	0.000
	5	A	0.144	0.027	5.288	0.000
	5	B	0.007	0.007	1.037	0.300
	5	C	2.787	0.757	3.684	0.000
	6	A	0.181	0.028	6.533	0.000
	6	B	-0.009	0.005	-1.680	0.093
	6	C	3.523	0.602	5.852	0.000
	7	A	0.173	0.019	9.009	0.000
	7	B	-0.009	0.003	-3.265	0.001
	7	C	3.267	0.314	10.392	0.000
2	4	A	0.074	0.039	1.897	0.058
	4	B	0.019	0.010	1.9497	0.051
	4	C	4.152	1.145	3.627	0.000
	5	A	0.099	0.027	3.633	0.000
	5	B	0.012	0.008	1.518	0.129
	5	C	4.202	1.221	3.443	0.001
	6	A	0.056	0.086	0.650	0.516
	6	B	0.000	0.000	1.049	0.294
	6	C	17.788	0.000	38076.98	0.000
	7	A	0.166	0.025	6.692	0.000
	7	B	-0.007	0.004	-2.078	0.038
	7	C	3.057	0.526	5.806	0.000
3	4	A	0.043	0.011	3.861	0.000
	4	B	0.063	0.005	13.502	0.000
	4	C	2.718	0.069	39.444	0.000
	5	A	0.0845	0.042	1.998	0.046
	5	B	0.011	0.009	1.119	0.263
	5	C	4.708	2.078	2.265	0.023
	6	A	-0.008	0.076	-0.101	0.919
	6	B	0.001	0.000	2.050	0.040
	6	C	17.752	0.001	23601.67	0.000
	7	A	0.176	0.028	6.275	0.000
	7	B	-0.009	0.004	-2.315	0.021
	7	C	3.101	0.465	6.666	0.000

Table A8: Quadratic fit to initial landing position curve (two-fixation cases) for reading ***mirrored-letter (mL)*** text: Estimates of parameters A, B and C

Session	Word Length	Parameters	Estimate	Std. Error	z value	Pr(z)
1	4	A	-0,007	0,022	-0,314	0.753
	4	B	0,078	0,009	8,332	0.000
	4	C	2,802	0,120	23,424	0.000
	5	A	0,009	0,017	0,494	0.621
	5	B	0,038	0,005	7,626	0.000
	5	C	3,593	0,172	20,842	0.000
	6	A	0,028	0,006	4,569	0.000
	6	B	0,016	0,001	12,389	0.000
	6	C	4,800	0,161	29,770	0.000
	7	A	0,015	0,016	0,943	0.346
	7	B	0,007	0,002	4,283	0.000
	7	C	6,717	0,787	8,540	0.000
2	4	A	0,002	0,004	0,446	0.656
	4	B	0,060	0,002	36,706	0.000
	4	C	3,150	0,035	89,4066	0.000
	5	A	1,316	0,331	3,974	0.000
	5	B	-0,002	0,001	-3,487	0.000
	5	C	-20,033	0,018	-1087,91	0.000
	6	A	0,002	0,019	0,095	0.924
	6	B	0,022	0,004	5,463	0.000
	6	C	4,544	0,324	14,023	0.000
	7	A	0,011	0,010	1,076	0.282
	7	B	0,010	0,002	6,450	0.000
	7	C	5,933	0,405	14,661	0.000
3	4	A	0,041	0,023	1,838	0.066
	4	B	0,054	0,010	5,436	0.000
	4	C	2,980	0,211	14,152	0.000
	5	A	0,010	0,021	0,487	0.631
	5	B	0,037	0,006	6,021	0.000
	5	C	3,654	0,227	16,110	0.000
	6	A	0,002	0,020	0,080	0.936
	6	B	0,024	0,004	5,568	0.000
	6	C	4,363	0,290	15,042	0.000
	7	A	-0,002	0,020	-0,123	0.902
	7	B	0,017	0,003	5,455	0.000
	7	C	4,990	0,329	15,169	0.000

Table A9: Quadratic fit to initial landing position curve (two-fixation cases) for ***reading inverted-word (iW)*** text: Estimates of parameters A, B and C

Session	Word Length	Parameters	Estimate	Std. Error	z value	Pr(z)
1	4	A	-0.460	0.116	-3.976	0.000
	4	B	0.002	0.000	5.742	0.000
	4	C	22.504	0.004	5642.92	0.000
	5	A	0.213	0.022	9.688	0.000
	5	B	-0.012	0.006	-1.884	0.060
	5	C	1.510	0.653	2.312	0.021
	6	A	0.214	0.026	8.132	0.000
	6	B	-0.018	0.005	-3.570	0.000
	6	C	2.940	0.243	12.093	0.000
	7	A	0.197	0.023	8.550	0.000
	7	B	-0.013	0.003	-3.954	0.000
	7	C	3.064	0.276	11.102	0.000
2	4	A	0.058	0.014	4.153	0.000
	4	B	0.030	0.005	5.758	0.000
	4	C	3.677	0.309	11.908	0.000
	5	A	0.006	0.054	0.115	0.909
	5	B	0.002	0.001	3.198	0.001
	5	C	11.017	0.001	11956.68	0.000
	6	A	0.042	0.116	0.361	0.718
	6	B	0.000	0.000	0.881	0.378
	6	C	24.665	0.001	41339.44	0.000
	7	A	0.167	0.025	6.785	0.000
	7	B	-0.008	0.004	-2.279	0.000
	7	C	3.522	0.439	8.024	0.000
3	4	A	0.021	0.010	2.219	0.000
	4	B	0.053	0.004	12.494	0.000
	4	C	3.185	0.106	30.049	0.000
	5	A	0.080	0.029	2.767	0.006
	5	B	0.014	0.008	1.763	0.078
	5	C	4.295	1.099	3.908	0.000
	6	A	0.057	0.114	0.502	0.616
	6	B	0.000	0.000	0.767	0.443
	6	C	25.887	0.000	51952.32	0.000
	7	A	0.165	0.022	7.515	0.000
	7	B	-0.008	0.003	-2.388	0.017
	7	C	3.685	0.426	8.654	0.000

Table A10: Quadratic fit to initial landing position curve (two-fixation cases) for reading ***scrambled-letter (sL)*** text: Estimates of parameters A, B and C

Session	Word Length	Parameters	Estimate	Std. Error	z value	Pr(z)
1	4	A	-0.034	0.034	-1.020	0.301
	4	B	0.087	0.014	6.051	0.000
	4	C	2.833	0.169	16.776	0.000
	5	A	0.017	0.008	2.123	0.034
	5	B	0.031	0.0024	13.118	0.000
	5	C	3.861	0.118	32.791	0.000
	6	A	0.022	0.008	2.859	0.004
	6	B	0.019	0.002	11.561	0.000
	6	C	4.563	0.155	29.519	0.000
	7	A	0.620	0.043	14.332	0.000
	7	B	0.000	7.177	-11.598	0.000
	7	C	-20.736	0.001	-19706.55	0.000
2	4	A	0.002	0.026	0.081	0.935
	4	B	0.086	0.010	8.373	0.000
	4	C	2.538	0.095	26.578	0.000
	5	A	-0.009	0.024	-0.363	0.716
	5	B	0.042	0.007	6.196	0.000
	5	C	3.580	0.210	17.015	0.000
	6	A	0.021	0.015	1.348	0.178
	6	B	0.014	0.003	4.705	0.000
	6	C	5.149	0.492	10.456	0.000
	7	A	0.618	0.055	11.303	0.000
	7	B	-0.001	0.000	-9.141	0.000
	7	C	-20.966	0.001	-17086.51	0.000
3	4	A	1.376	0.758	1.815	0.070
	4	B	-0.002	0.001	-1.553	0.121
	4	C	-22.215	0.041	-547.09	0.000
	5	A	0.025	0.009	2.778	0.005
	5	B	0.029	0.003	10.999	0.000
	5	C	3.918	0.145	27.020	0.000
	6	A	0.020	0.020	0.984	0.325
	6	B	0.015	0.004	3.698	0.000
	6	C	5.046	0.601	8.399	0.000
	7	A	-0.027	0.011	-2.442	0.015
	7	B	0.003	0.000	16.467	0.000
	7	C	10.061	0.000	49984.09	0.000

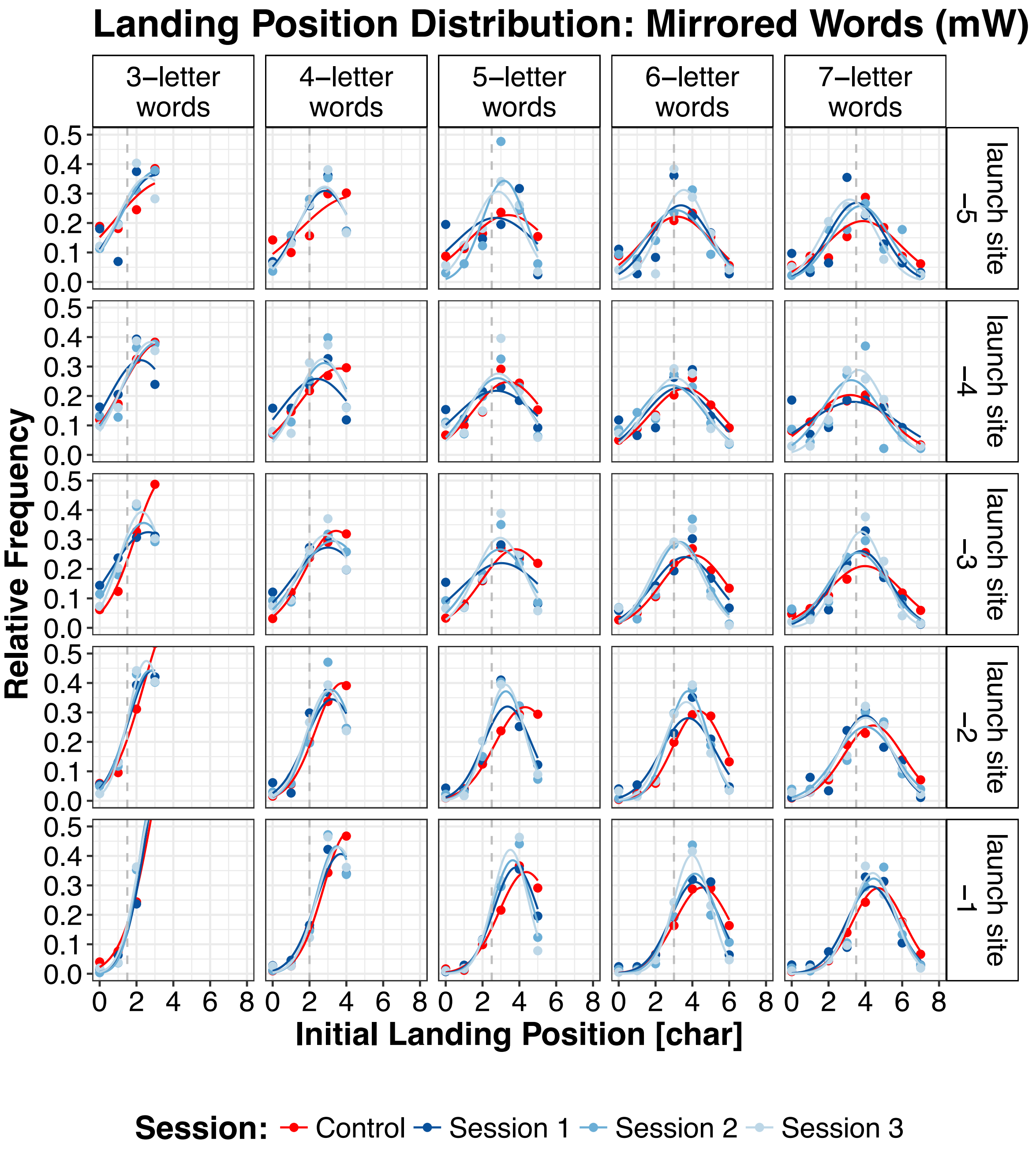

Figure F1: Within-word landing position distributions grouped by launch-site distance and word length for reading *mirrored-word (mW)* texts. Red line and dots represent data from normal reading session. Data from the first experimental session are presented in dark blue color. The lighter blue hues represent the last two experimental sessions.

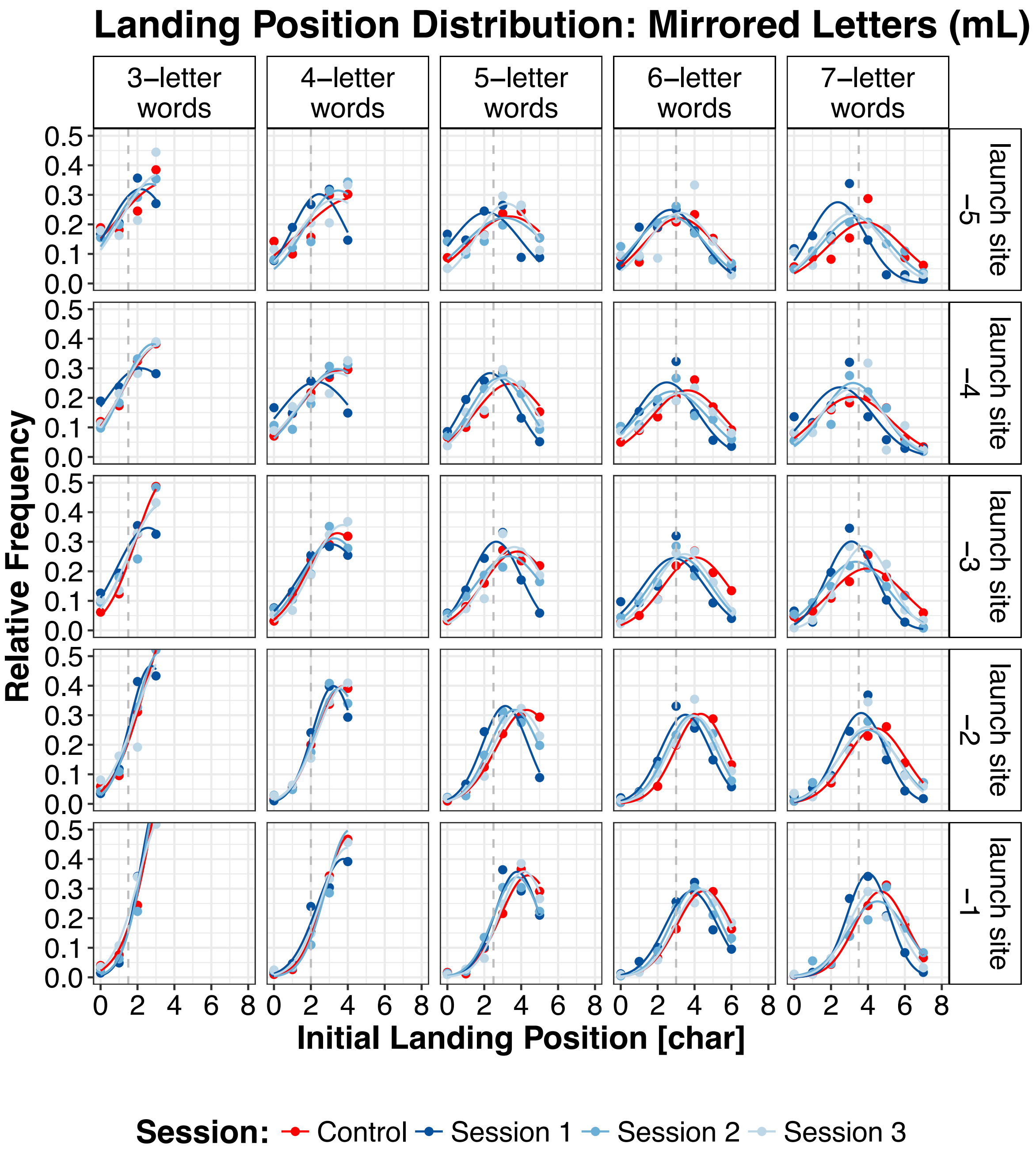

Figure F2: Within-word landing position distributions grouped by launch-site distance and word length for reading *mirrored-letter (mL)* texts. Red line and dots represent data from normal reading session. Data from the first experimental session are presented in dark blue color. The lighter blue hues represent the last two experimental sessions.

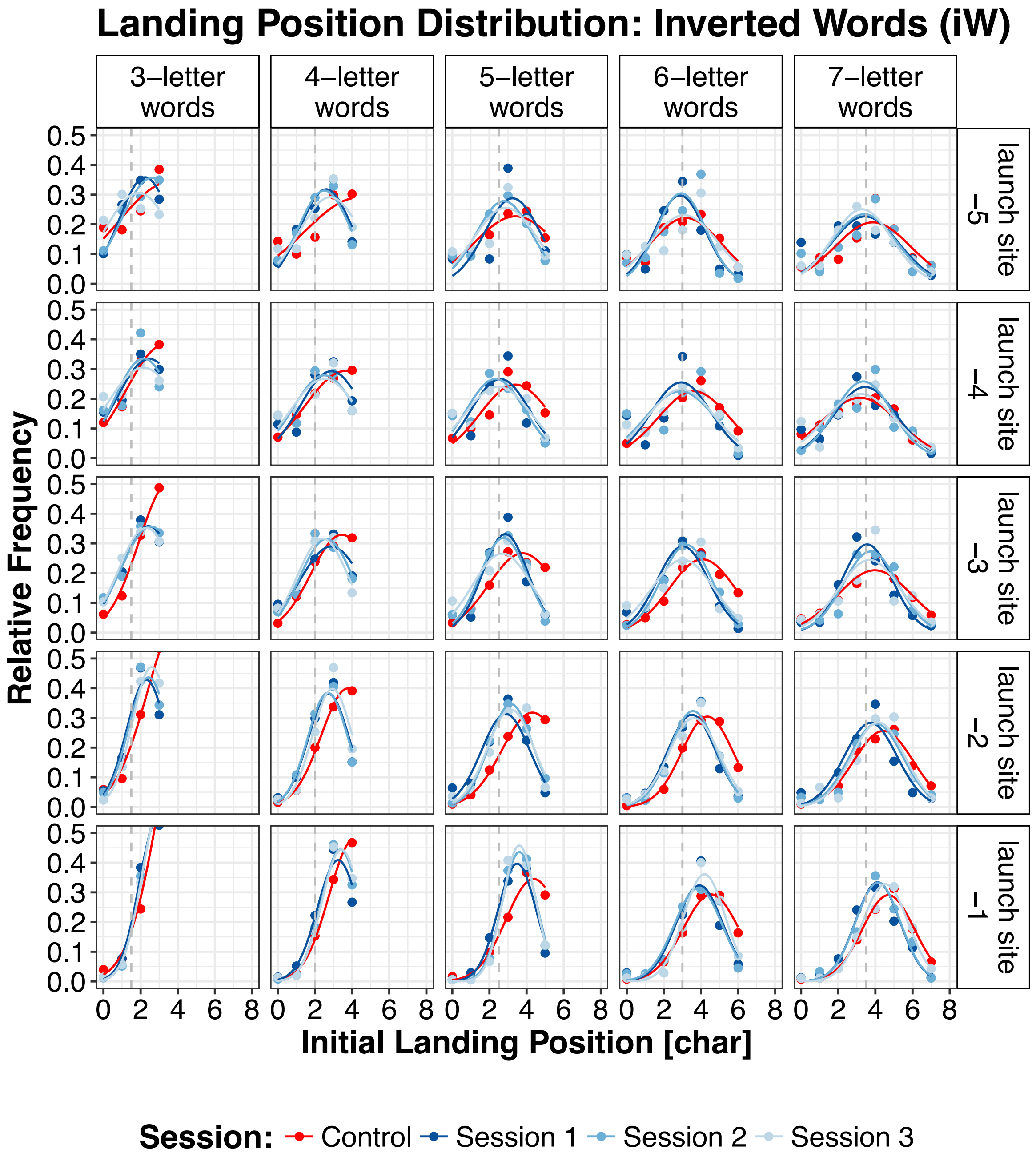

Figure F3: Within-word landing position distributions grouped by launch-site distance and word length for reading *inverted-word (iW)* texts. Red line and dots represent data from normal reading session. Data from the first experimental session are presented in dark blue color. The lighter blue hues represent the last two experimental sessions.

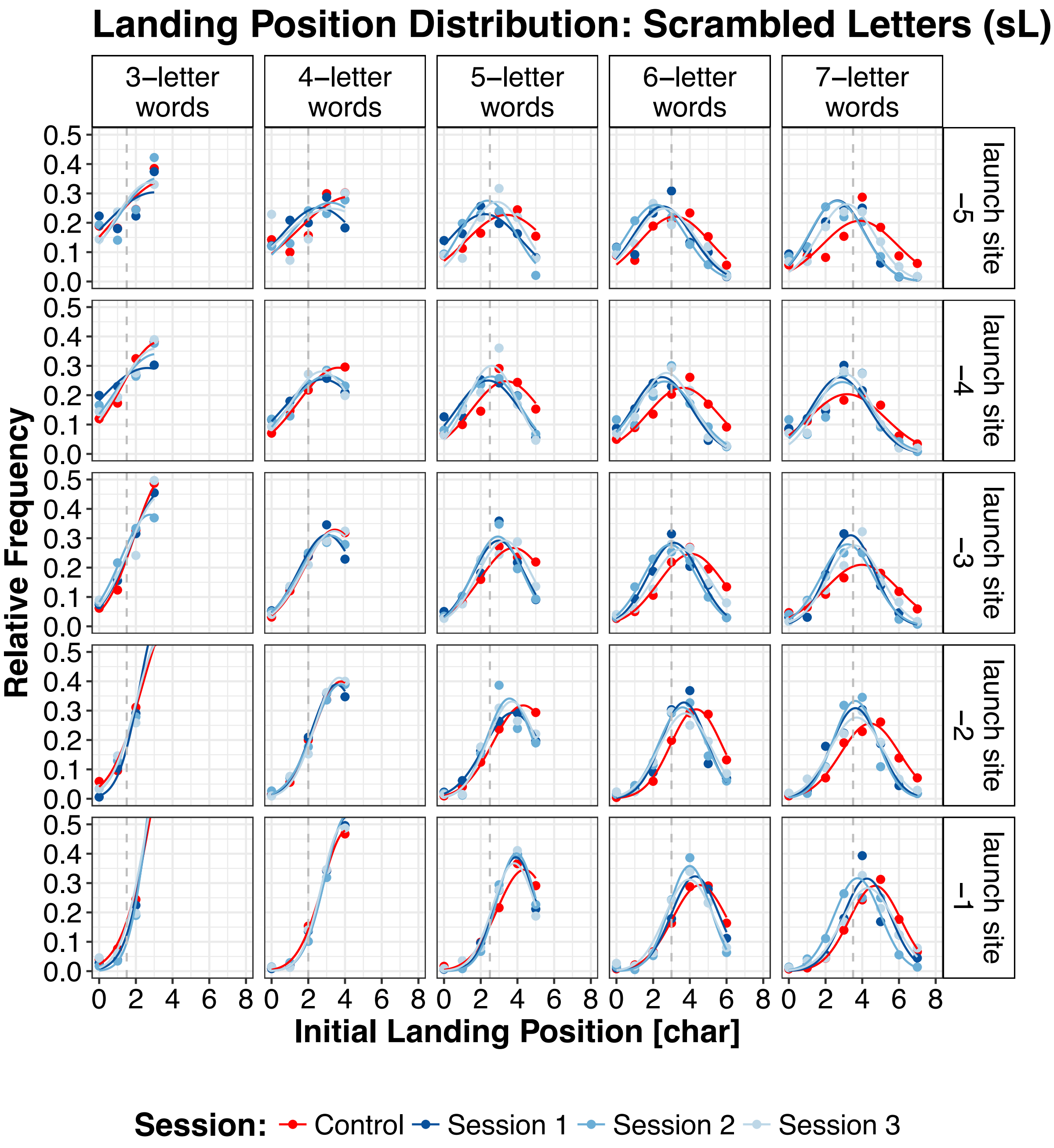

Figure F4: Within-word landing position distributions grouped by launch-site distance and word length for reading *scrambled-letter (sL)* texts. Red line and dots represent data from normal reading session. Data from the first experimental session are presented in dark blue color. The lighter blue hues represent the last two experimental sessions.

References

Bates, D., Mächler, M., Bolker, B., & Walker, S. (2015). Fitting linear mixed-effects models using lme4. *Journal of Statistical Software*, *67*(1), 1-48. doi: 10.18637/jss.v067.i01

Engbert, R., & Nuthmann, A. (2008, 02). Self-consistent estimation of mislocated fixations during reading. *PLOS ONE*, *3*(2), 1-6. Retrieved from `https://doi.org/10.1371/journal.pone.0001534` doi: 10.1371/journal.pone.0001534

Kruschke, J. K. (2014). *Doing bayesian data analysis: A tutorial with r, jags, and stan.* Academic Press.

McConkie, G. W., Kerr, P. W., Reddix, M., & Zola, D. (1988). Eye movement control during reading: I. the localisation of initial eye fixations in words. *Visual Research*, *28*(10), 1107-1118. doi: https://doi.org/10.1016/0042-6989(88)90137-X

Plummer, M. (2016). rjags: Bayesian graphical models using mcmc [Computer software manual]. Retrieved from `https://CRAN.R-project.org/package=rjags` (R package version 4-6)
